# Ileo-colic intra-corporeal anastomosis during robotic right colectomy: a systematic literature review and meta-analysis of different techniques

**DOI:** 10.1007/s00384-021-03850-9

**Published:** 2021-01-23

**Authors:** Simone Guadagni, Matteo Palmeri, Matteo Bianchini, Desirée Gianardi, Niccolò Furbetta, Fabrizio Minichilli, Gregorio Di Franco, Annalisa Comandatore, Giulio Di Candio, Luca Morelli

**Affiliations:** 1 General Surgery Unit, Department of Translational Research and new Technologies in Medicine and Surgery, University of Pisa, Via Paradisa 2, 56124 Pisa, Italy; 2 Unit of Environmental Epidemiology and Disease Registries, Institute of Clinical Physiology, National Council of Research, Pisa, Italy; 3 Endo-CAS (Center for Computer Assisted Surgery), University of Pisa, Pisa, Italy

**Keywords:** Robotic surgery, Intra-corporeal anastomosis, Right colectomy, da Vinci

## Abstract

**Purpose:**

Robotic assistance could increase the rate of ileo-colic intra-corporeal anastomosis (ICA) during robotic right colectomy (RRC). However, although robotic ICA can be accomplished with several different technical variants, it is not clear whether some of these technical details should be preferred. An evaluation of the possible advantage of one respect to another would be useful.

**Methods:**

We conducted a systematic review of literature on technical details of robotic ileo-colic ICA, from which we performed a meta-analysis of clinical outcomes. The extracted data allowed a comparative analysis regarding the outcome of overall complication (OC), bleeding rate (BR) and leakage rate (LR), between (1) mechanical anastomosis with robotic stapler, versus laparoscopic stapler, versus totally hand-sewn anastomosis and (2) closure of enterocolotomy with manual double layer, versus single layer, versus stapled.

**Results:**

A total of 30 studies including 2066 patients were selected. Globally, the side-to-side, isoperistaltic anastomosis, realized with laparoscopic staplers, and double-layer closure for enterocolotomy, is the most common technique used. According to the meta-analysis, the use of robotic stapler was significantly associated with a reduction of the BR with respect to mechanical anastomosis with laparoscopic stapler or totally hand-sewn anastomosis. None of the other technical aspects significantly influenced the outcomes.

**Conclusions:**

ICA fashioning during RRC can be accomplished with several technical variants without evidence of a clear superiority of anyone of these techniques. Although the use of robotic staplers could be associated with some benefits, further studies are necessary to draw conclusions.

## Introduction

The application of minimally invasive surgery in colorectal procedures has rapidly spread worldwide. In particular, laparoscopic right colectomy is associated with earlier return to normal bowel function, shorter length of hospital stay, fewer wound complications and similar oncological outcomes respect to the conventional open approach [1]. Thus, minimally invasive right colectomy is nowadays a commonly performed procedure.

However, in spite of the well-demonstrated benefits of the complete intra-corporeal anastomosis (ICA), such as the faster bowel recovery and less analgesic usage, thanks to the reduced mesenteric traction, with respect to the extracorporeal technique [2, 3], its widespread usage during a minimally invasive right colectomy is still limited.

Indeed, due to the laparoscopic skills required, especially in terms of suturing, it is estimated that less than 10% of procedures are accomplished with the fashioning of ileo-colic ICA [4] and most surgeons performing laparoscopic right colectomy still use the extracorporeal approach.

In this context, the application of robotic surgery has been considered an appealing advancement. The da Vinci robotic system has gained popularity in colorectal surgery because it has been expected to overcome the steering learning curve of laparoscopy and to allow an easier access to narrow spaces. A lot of papers have shown a reduction of conversion rates also in ‘difficult cases’ [5]. Moreover, thanks to the introduction of the da Vinci Xi platform, together with its specific tools, the two main drawbacks of robotic surgery, operative time and costs, seem to be flattened [6, 7]. Recently, the attention has been focused on right colectomy with the introduction of robotic ‘top to down’ complete mesocolic excision and the sovra-pubic approach [8, 9]. In all these surgical procedures, robotic assistance may decrease workload and improve suturing performance. Therefore, by overcoming the kinematic limitations of pure laparoscopy, it could play a key role also in increasing the adoption rate of ICA during robotic right colectomy (RRC).

Similarly to laparoscopy, robotic ICA can be accomplished with several different technical variants, such as, for instance, mechanical or totally hand-sewn, single- or double-layered, and by using several types of suture. As until now it is not clear if some of these technical details should be preferred, we aimed to provide a systematic review of the literature about ICA during RRC and to compare the different anastomosis variants evaluating intra- and post-operative outcomes, in order to find if one of these surgical techniques may be superior to the others.

## Methods

An extensive literature review from inception to March 2020 using PubMed database for English literature was performed. Research question was are there any differences in terms of clinical outcomes between the several technical variants of robotic intracorporeal ileo-colic anastomosis fashioning? The searched formulas were ‘Robotic AND intra-corporeal anastomosis’, ‘Robotic AND ileo-colic anastomosis’, ‘Robotic AND right colectomy’, ‘Robotic AND complete mesocolic excision’, ‘Robotic AND transverse colon’.

**Fig. 1:**
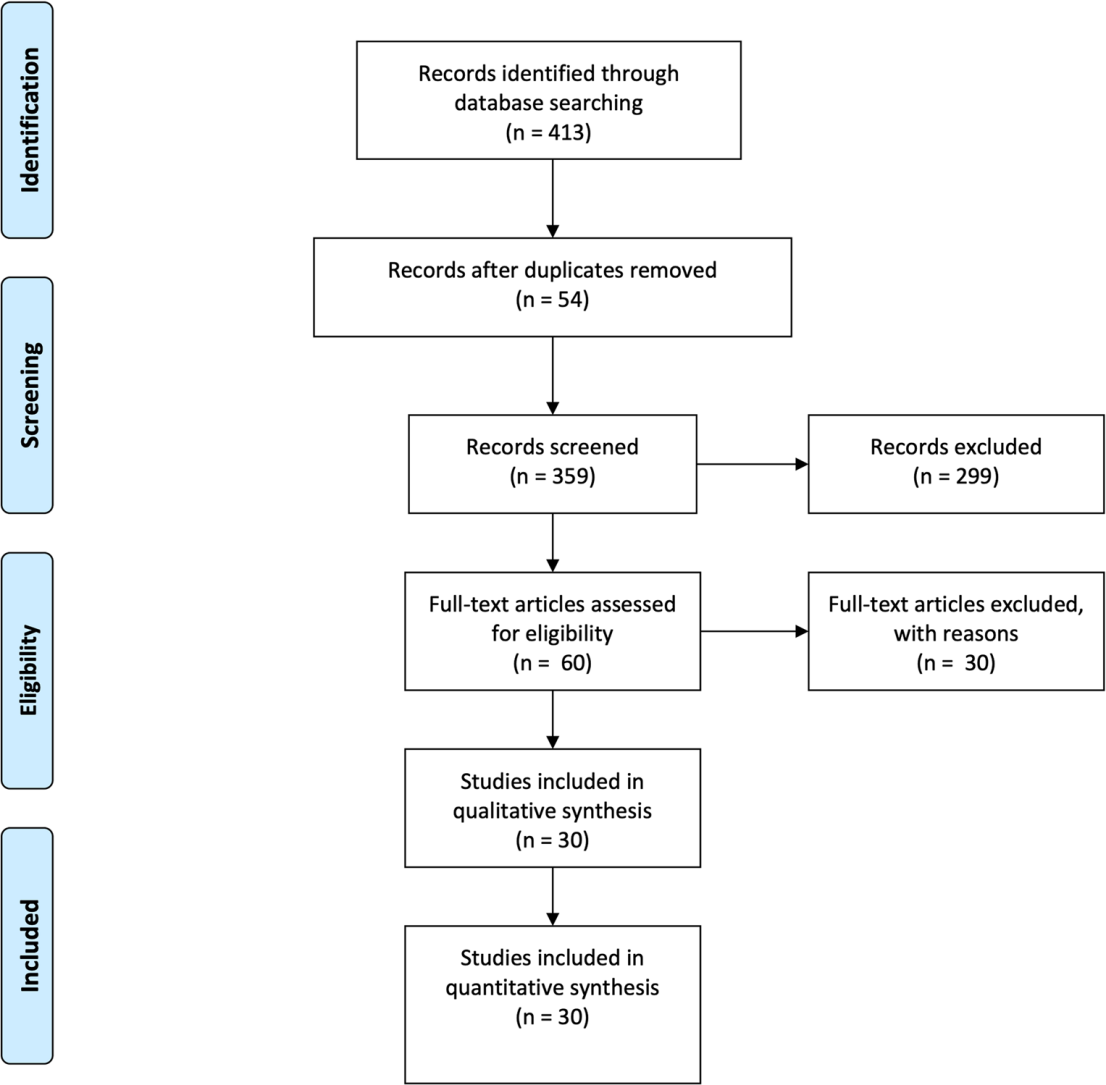
PRISMA diagram of literature research

Article selection was carried out according to the preferred reporting items for systematic reviews and meta-analyses criteria (PRISMA) [10] (Fig. 1) and AMSTAR (assessing the methodological quality of systematic reviews) guidelines. Manuscripts identified by cross-referencing were also retrieved and evaluated. Inclusion criteria were as follows: articles in English, reporting more than 10 robot-assisted right colectomies with ileo-colic ICA and in which the authors provided a detailed description of their robotic technique. Exclusion criteria were as follows: original articles with less than 10 patients, case reports, letters to the editor, editorial comments or other works without clinical records or without technical description of the anastomosis.

From articles, comparing robotic extra- and ICA, we extracted only technical and clinical data of the ICA group. In the same manner, from studies comparing laparoscopic and RRC, we extracted only technical and clinical data of the robotic group.

We extracted from each study the following data: number of patients, mean age, male/female ratio, type of anastomosis, stapler used, opening and closure of enterotomies. The suture type, the stapler and the cartridge adopted were also retrieved. Finally, the complication rate was recorded with attention to overall complications (OC), anastomotic leakage and bleeding. OC was defined as overall post-operative complications, both medical and surgical following the Clavien-Dindo classification [11], and reported by the authors. Leakage rate (LR) was defined as any enteral leak from ICA that was treated conservatively or that required any interventional procedure in the post-operative course, and reported by the authors. Bleeding rate (BR) was defined as any intraluminal haemorrhage from the anastomotic site that was treated conservatively or that required any interventional procedure in the post-operative course, and reported by the authors.

Other details not specifically inherent to the ICA technique, such as pre-operative diagnosis, histopathologic diagnosis, number of lymph nodes harvested, follow-up beyond the post-operative course, were not extracted in the present review because not useful for the purpose of this study.

From the systematic review, we also performed a meta-analysis of clinical outcomes, including all patients of those articles for which it was possible to obtain adequate data for a statistically significant analysis, and for which all the details were clearly deducible.

Three authors (SG, MB and NF) independently reviewed all the manuscripts that met the inclusion criteria. The final search was completed by April 15, 2020. This systematic review and meta-analysis has been registered on PROSPERO [registration number CRD42020213777].

### Statistical analysis

Meta-analysis was performed using the ‘metaprop’ routine [12] by the Stata statistics software (version 15 for Windows Stata Corporation, 2017). The metaprop routine entails the Freeman-Tukey double arcsine transformation procedure and DerSimonian-Laird random-effects model [13, 14]. Specifically, the Freeman-Tukey double arcsine procedure transforms proportions from individual studies by stabilizing between-study variance. Subsequently, the DerSimonian-Laird random-effects model computes the weighted overall pooled estimates. Between-study heterogeneity was assessed by inspecting the forest plots and the chi-squared test for heterogeneity. *I*^2^ statistic with a value above 50% was interpreted as representing high heterogeneity [15], and therefore, a random-effects model analysis was used. When heterogeneity was modest (*I*^2^ < 50%), a fixed-effects model of analysis was performed. Results of the meta-analysis were reported as pooled prevalence of OC, LR and BR with 95% confidence intervals (CIs); *p* values < 0.05 were considered statistically significant.

## Results

### Descriptive analysis

Using the search terms listed above, 413 publications were identified. No randomized trial was found. After title and abstract review, 353 articles were excluded as duplicate or non-pertinent. The remaining 60 studies were investigated in detail. Thirty of them were than excluded for the following reasons: articles without clinical records (*n* = 10), case reports (*n* = 14), case series with less than 10 patients involved (*n* = 6). Then, we finally selected 30 studies [8, 15–43] involving a total of 2066 patients (Table 1). The only article that specifically compared different techniques used during minimally invasive ICA (either robotic and laparoscopic) was conducted by Milone et al. and was included in our review for the robotic part.

**Table 1:** Characteristics of the studies included in the systematic review and analysis

Studyes (year of publication)	# of patients	Gender M/F	Age Mean	Type of anastomosis	Stapler used	Opening of EC	Closure of EC	Complications (range)
Scotton (2018) [16] Kelley (2018) [17] Hamzaoglu (2018) [8] Akram (2018) [18]	357	181/176	68	Mechanical Isoperistaltic	Robotic blue load (1)	Monopolar scissor	CDL 3/0 Assufil, Quill, V-loc	OC 14–44% Leak 0–0.5% Bleeding 0–5%
Ozben (2019) [19]	37	20/17	64	Mechanical Isoperistaltic	Robotic	Monopolar scissor	Continuous V-loc 3/0 IL; interrupted silk 3/0 SdL	OC 21% Leak 0% Bleeding 2.7%
Blumberg (2018) [20]	21	7/14	65	Mechanical Isoperistaltic	Robotic blue load	Monopolar scissor	Stapled	OC 14% Leak 4.7% Bleeding 0%
Johnson (2019) [21] Ozoben (2018) [22]	125	56/69	64	Mechanical Isoperistaltic	Robotic blue load (1)	NS	CSL	OC 0–7.4% Leak 0% Bleeding 0–1%
Mégevand (2019) [23] Yozgatli (2019) [24] Bae (2019) [25] Spinoglio (2019) [27] Raimondi (2018) [26] Lujan (2018) [28] Ngu (2018) [29] Petz (2017) [30] Parisi (2017) [31] Spinoglio (2016) [32] Morpurgo (2013) [33] Lujan (2013) [34]	654	351/303	69	Mechanical Isoperistaltic	Laparoscopic 60 mm (5) or 45 (1)	Monopolar scissor (2), hook (1), Harmonic scalpel (1)	CDL 2/0 Vicryl (1), Barbed suture / V-loc (3)	OC 4–31% Leak 0–4% Bleeding 0–10%
Ioannidis (2018) [35] Trastulli (2013) [36]	65	28/37	64	Mechanical Isoperistaltic	Laparoscopic 60 mm (1)	Monopolar hook (1)	Continuous Barbed 2/0 or PDS IL; interrupted PDS 2/0 SdL	OC 4–5% Leak 0% Bleeding 0%
Reitz (2018) [37]	29	11/28	60	Mechanical Isoperistaltic	Laparoscopic EndoGIA 60 mm TriStaple™	Monopolar scissor	Stapled	OC 9% Leak 0% Bleeding 3%
Milone (2019) [38] Cleary (2018) [39]	512	282/230	68	Mechanical Variable	Laparoscopic	NS	Variable (DL)	OC 15% Leak 1.7% Bleeding 4–8.5%
Liu (2019) [40] Park (2012) [41] Park (2012) [42]	114	54/60	61	Mechanical Antiperistaltic	Laparoscopic	Monopolar scissor	Stapled	OC 17–33% Leak 0–2.8% Bleeding 0–2.8%
Trastulli (2015) [43] D’Annibale (2010) [44]	152	80/	71	Hand-sewn Isoperistaltic	Not used	Not used	Hand-sewn SL (1), DL (2) absorbable 3/0 monofilament	OC 1.3–26% Leak 0–2.9% Bleeding 0–2%

*CDL* continuous double layer, *IL* inner layer, *SdL* second layer, *NS* not specified, *CSL* continuous single layer, *SL* single layer, *DL* double layer, *OC* overall complications

In eight manuscripts, the robotic platform used was the da Vinci Xi (Intuitive Surgical Inc., Sunnyvale, CA, USA); in six manuscripts, both the da Vinci Xi and the da Vinci Si were used, whereas in twelve manuscripts, the da Vinci Si was the only robotic system considered. In four papers, the specific robotic platform used was not mentioned.

Eight articles [8, 16–22] described the use of robotic stapler in fashioning an ileo-colic anastomosis. All the anastomoses were mechanical isoperistaltic. The type of cartridge was specified only in three articles (Kelly et al. [17], Blumberg et al. [20] and Johnson et al. [21]), with the blue load used for all of them. The main difference arisen from these studies concerns the closure of the enterocolotomy. Five studies described a double-layer closure: Scotton et al. [16] used a 3/0 Assufil, Kelly et al. [17] a 3/0 Quill suture, whereas Hamzaoglu et al. [8] used 3/0 V-Loc suture. Ozben et al. [19] closed the enterocolotomy in a double layer but they preferred continuous 3/0 V-Loc for the inner layer and interrupted 3/0 silk for the second one. Instead, two manuscripts reported a single layer continuous suture in this phase, without specifying the type of suture [21, 22]. Only Blumberg et al. [20] in 2018 closed the defect with another firing of robotic 3.5 mm stapler.

The use of a laparoscopic stapler was reported in twenty articles [23–42] with only two manuscripts (Bae et al. [25] and Petz et al. [30]) describing the use of both robotic and laparoscopic staplers.

The mechanical isoperistaltic orientation for ICA fashioning was reported in fifteen articles [23–37] and it was the most used one in the two multi-center trials (performed by Milone et al. [38] and Cleary et al. [39]). Anti-peristaltic orientation was reported only by three articles [40–42].

Considering the type of laparoscopic stapler, in two articles, a 45 mm Echelon Endopath was used; in five articles, the mechanical anastomosis was performed with 60 mm Echelon Endopath (with the blue load described in two articles). Only in one case series, the anastomosis was created with EndoGIA Tri-Stapler purple load. In the remaining studies, the type of laparoscopic stapler used was not mentioned. The closure of enterocolotomy was performed with double layer 2/0 Vicryl by Lujan et al. [28], while double layer with barbed/self-anchoring sutures was described by Yozgatli et al. [24], Bae et al. [25] and Raimondi et al. [26] respectively. Ioannidis et al. [35] used a continuous barbed suture for the inner layer and an interrupted 2/0 PDS for the second layer, while Trastulli et al. [36] used a continuous 2/0 PDS suture for the inner layer and an interrupted 2/0 PDS for the second layer. In the remaining eight studies, the type of suture used for double-layer closure of enterocolotomy was not mentioned.

Only in two manuscripts, a totally hand-sewn ICA after right colectomy was considered by Trastulli et al. [43] and D’Annibale et al. [44]. The first authors used a single layer of 3/0 absorbable monofilament suture; meanwhile, D’Annibale et al. performed it with a double layer of 3–0 absorbable monofilament.

The extracted data allowed a statistically significant comparative analysis regarding the outcome of OC, bleeding rate (BR) and leakage rate (LR), between (1) mechanical anastomosis with robotic stapler, versus laparoscopic stapler, versus totally hand-sewn anastomosis and (2) closure of enterocolotomy with manual double layer, versus manual single layer, versus stapled.

It was not possible to compare the type of suture used or sutures’ characteristics (such as running or interrupted sutures), due to the small number and the high heterogeneity of the reported data on articles that specifically described these aspects. For the same reasons, differences in cartridge load during robotic or laparoscopic mechanical anastomosis were not statistically analysed.

## Meta-analysis

### Mechanical anastomosis with robotic stapler, versus laparoscopic stapler, versus totally hand-sewn anastomosis

A total of thirty articles comprising 2066 patients were considered. The mechanical anastomosis with robotic stapler group (MC Rob group) included eight studies with a total of 540 patients involved, whereas the mechanical anastomosis with laparoscopic stapler group (MC Lap group) included twenty studies with a total of 1374 patients involved. Finally, totally hand-sewn anastomosis group (TS group) included two studies with a total of 152 patients. Leakage and bleeding rates were retrieved from all articles, while OC was retrieved from twenty-seven manuscripts (96% of total).

### Pooled prevalence of OC

**Fig. 2:**
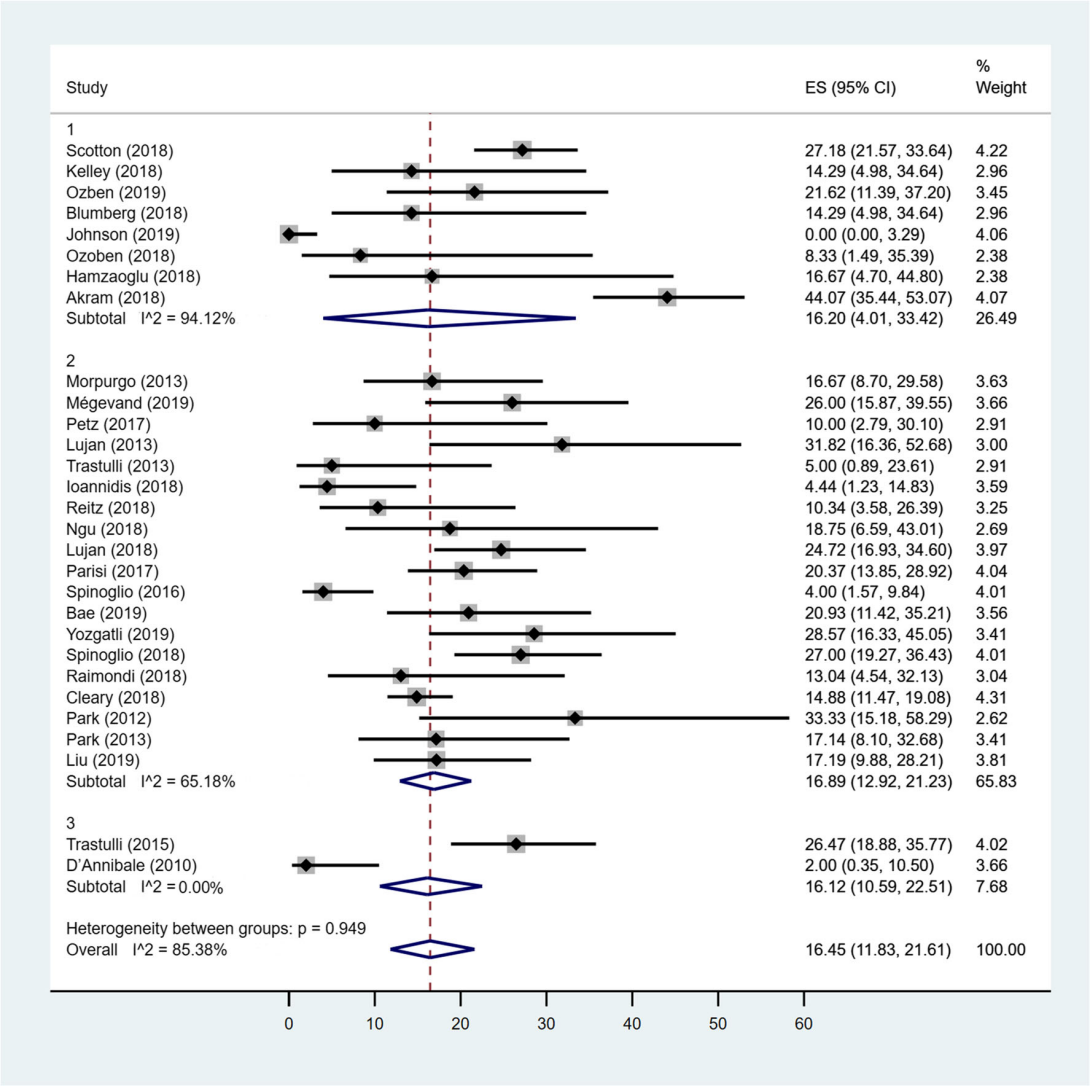
Forest plot showing OC in MC Rob group (1), MC Lap group (2), TS group (3) and overall studies

The estimated overall pooled prevalence of OC was 16.45% (95% CI 11.83–21.61). The pooled prevalence of OC for the MC Rob group was 16.20% (95% CI .01–33.42), the pooled prevalence of OC among patients in MC Lap group was 16.89% (95% CI 12.92–21.23), while the prevalence among patients in TS group was 16.12% (95% CI 10.59–22.51). Studies conducted in MC Rob group had a considerably higher heterogeneity (*I*^2^ = 94.12%) than those in Lap group (*I*^2^ = 65.18%). The difference between the three groups was not statistically significant (*p* = 0.949) (Fig. 2).

### Pooled prevalence of LR

**Fig. 3:**
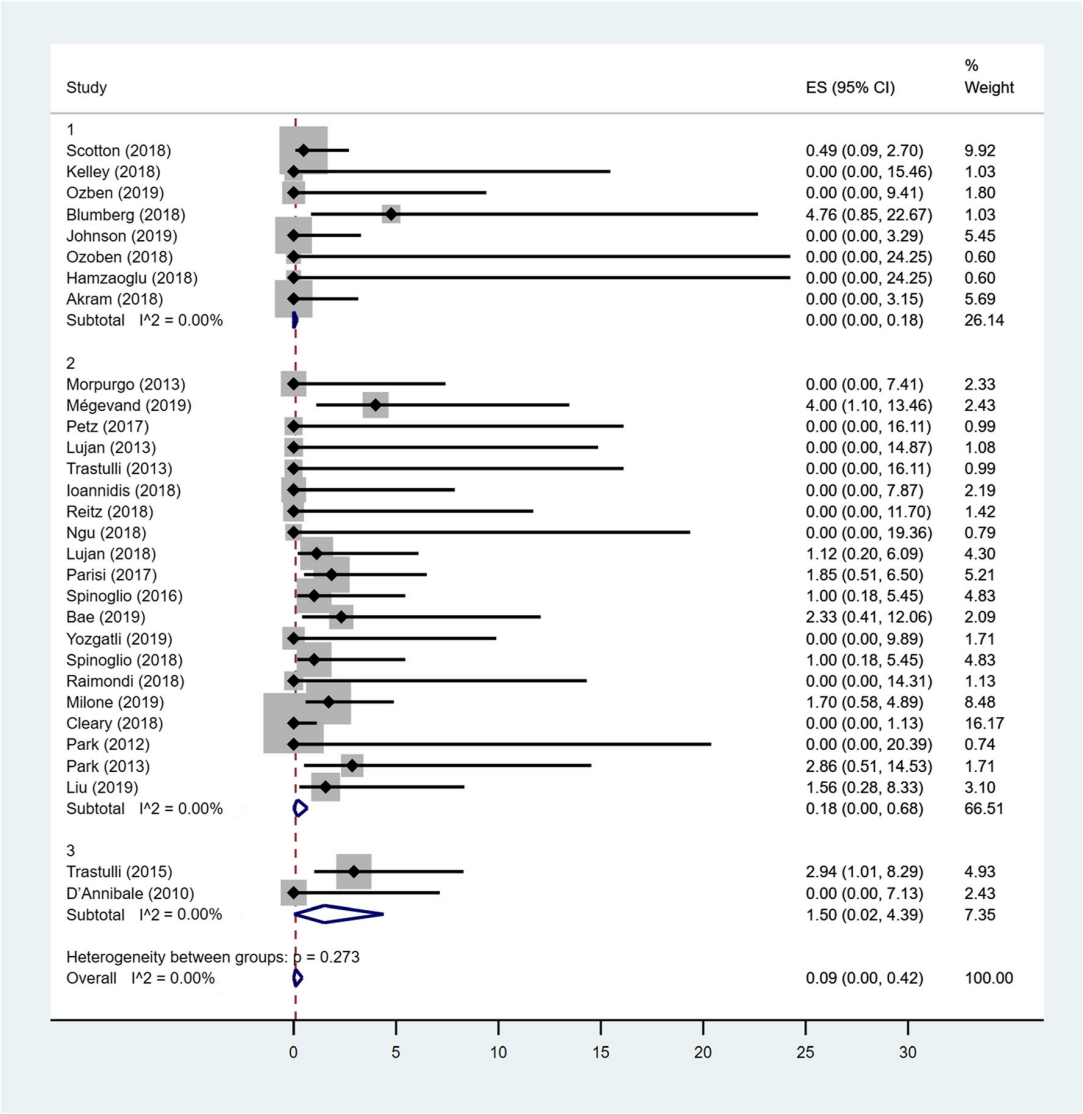
Forest plot showing leakage rate in MC Rob group (1), MC Lap group (2), TS group (3) and overall studies

Considering all groups, no heterogeneity between studies was detected. The fixed estimate overall pooled prevalence of leakage was 0.09% (95% CI 0–0.42). The estimated pooled prevalence of leakage for MC Rob and MC Lap groups was 0% (95% CI 0–0.18) and 0.18% (95%CI 0–0.68) respectively, while in TS group, the prevalence was 1.50% (95% CI 0.02–4.39). The difference between all groups was not statistically significant (*p* = 0.273) (Fig. 3).

### Pooled prevalence of BR

**Fig. 4:**
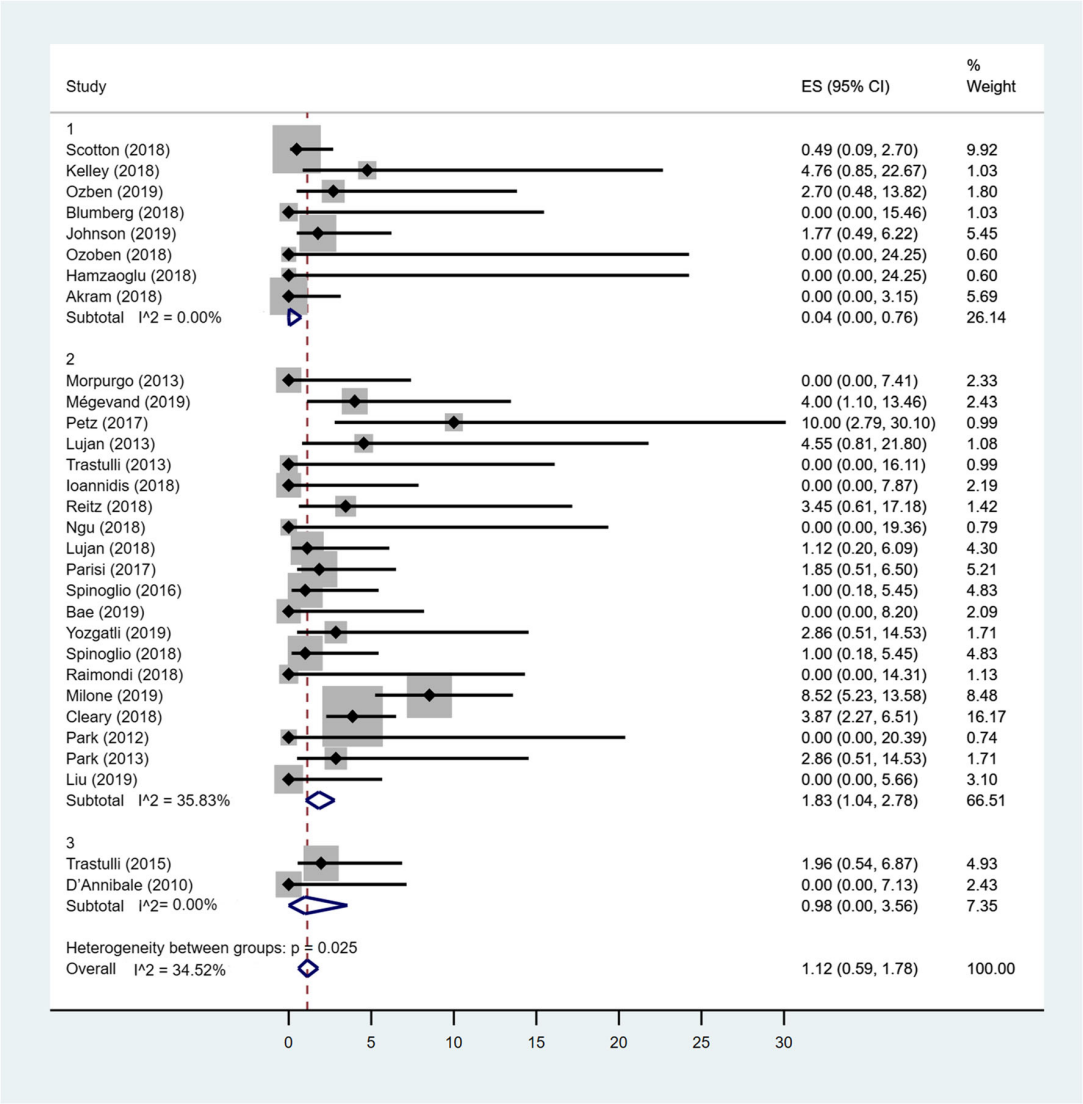
Forest plot showing bleeding rate in MC Rob group (1), MC Lap group (2), TS group (3) and overall studies

The overall fixed-effects pooled prevalence of bleeding was 1.12% (95% CIs: 0.59–1.78) with a low heterogeneity between studies (*I*^2^ = 34.52%). When only MC Lap group was considered, the pooled prevalence increased to 1.83% (95% CIs: 1.04–2.78; heterogeneity *I*^2^ = 35.83%). Considering both MC Rob group and TS groups, no heterogeneity between studies resulted (*I*^2^ = 0%). The fixed-effects pooled prevalence of MC Rob group were 0.04% (95% CIs: 0–0.76). For TS group, the pooled prevalence was 0.98% (95% CIs: 0–3.56). The difference between groups was statistically significant (*p* = 0.026) with a lower BR in the MC Rob group (0.04%) with respect to MC Lap group (1.83%) and to TS group (0.98%) (Fig. 4).

## Closure of enterocolotomy with double layer, versus single layer, versus stapled

A total of twenty-six articles with 1402 patients were considered. We excluded from this analysis the studies conducted by Milone et al. and Cleary et al., as we cannot define exactly the total number of patients in which the closure of enterocolotomy during robotic mechanical ICA was performed in a single or double layer. The first group, mechanical ICA with double-layer closure of enterocolotomy (MD group), comprised nineteen articles with a total of 1113 patients; the second group, mechanical ICA with single layer closure of enterocolotomy (MS group), included two papers with a total of 125 patients. The third group, mechanical ICA with stapled closure of enterocolotomy (MSt group), included five articles with a total of 164 patients.

### Pooled prevalence of OC

**Fig. 5:**
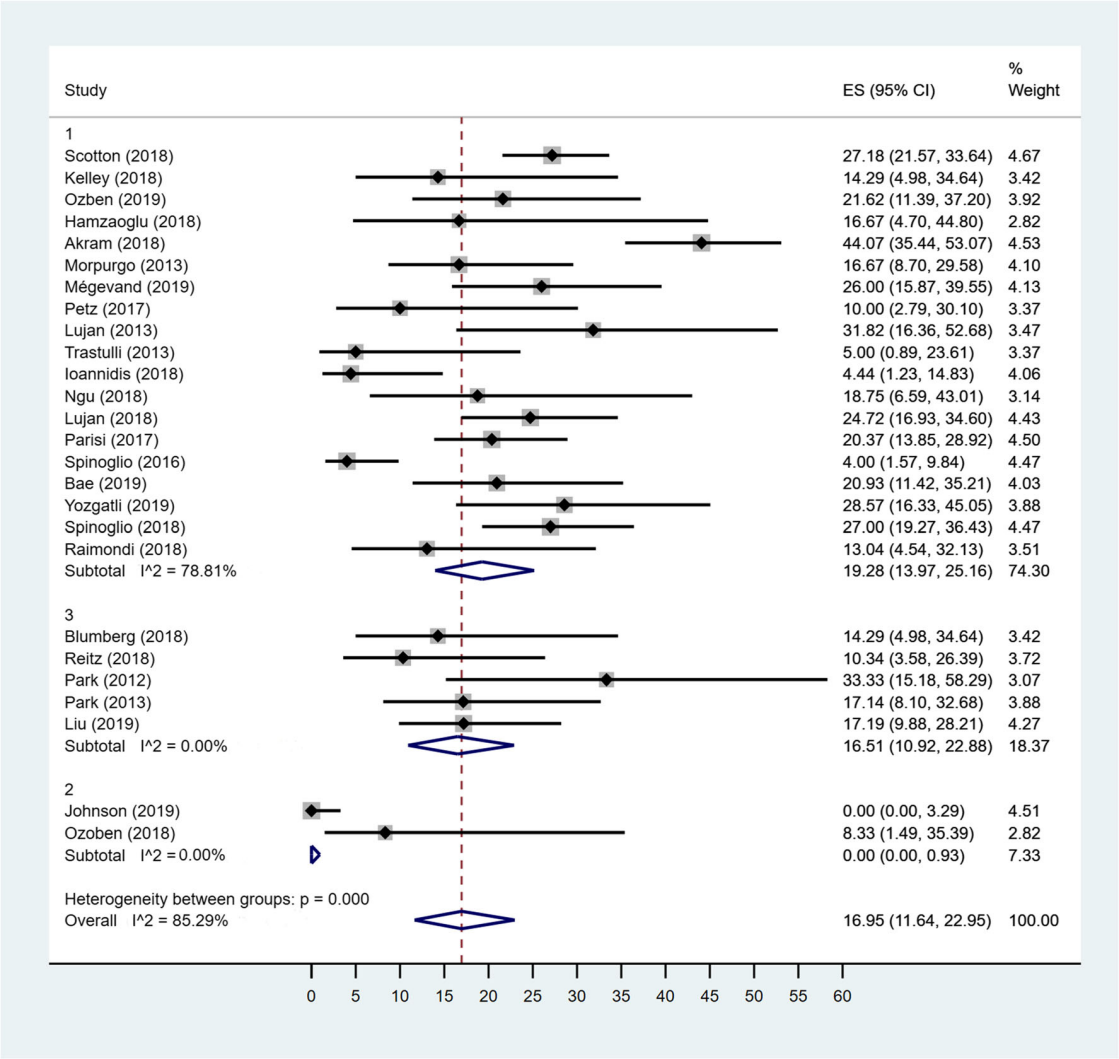
Forest plot showing OC in MD group (1), MS group (2), MSt group (3) and overall studies

The overall random-effects pooled prevalence of OC was 16.95% (95% CIs: 11.64–22.95) with a high level of heterogeneity (*I*^2^ = 85.29%) (Fig. 5). When only studies concerning MD group were considered, the pooled prevalence increased to 19.28% (95% CIs: 13.97–25.16), with reduced, but still high, heterogeneity (*I*^2^ = 78.81%). For both MSt and MS groups, the *I*^2^ was 0%; the MSt pooled prevalence of OC was 16.51% (95% CIs: 10.92–22.88) and for MS group, the pooled prevalence was 0% (95% CIs: 0–0.93). The difference between groups was statistically significant (*p* < 0.001) with a higher OC in the MD group (19.28%) and MSt group (16.51%) with respect to MS group (0%) (Fig. 5).

### Pooled prevalence of LR

**Fig. 6:**
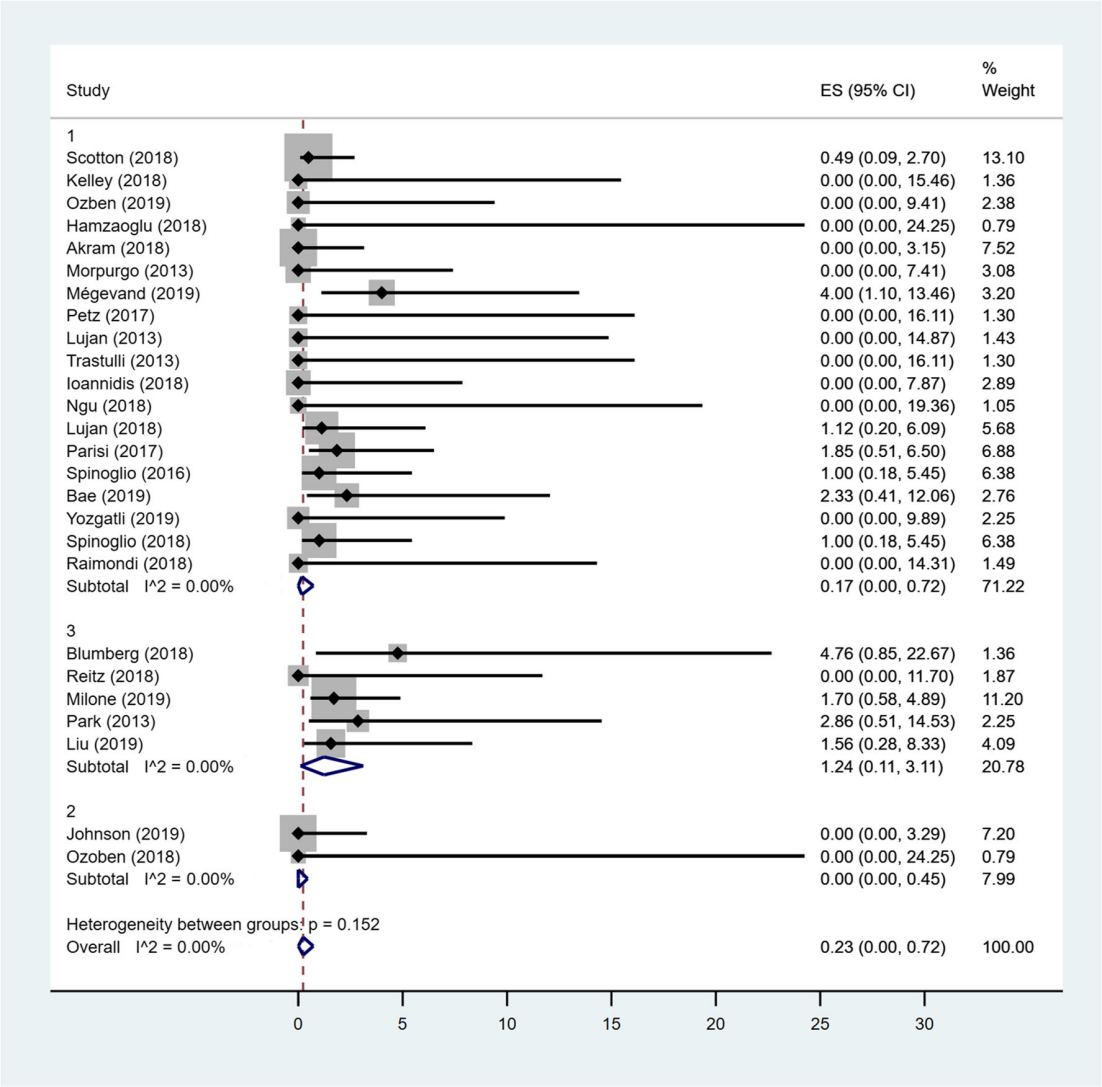
Forest plot showing leakage rate in MD group (1), MS group (2), MSt group (3) and overall studies

Considering that in all groups, there was no heterogeneity between studies (*I*^2^ = 0); then, the fixed effects model was used to estimate the pooled prevalence. The overall pooled prevalence of leakage was 0.23% (95% CI 0–0.72). The pooled prevalence of leakage for the MD group was 0.17% (95% CI 0–0.72).The pooled prevalence of leakage for the MSt group was 1.24% (95% CI 0.11–3.11), while the prevalence among patients in MS groups was 0% (95% CI 0–0.45). The difference between groups was not statistically significant (*p* = 0.152) (Fig. 6).

### Pooled prevalence of BR

**Fig. 7:**
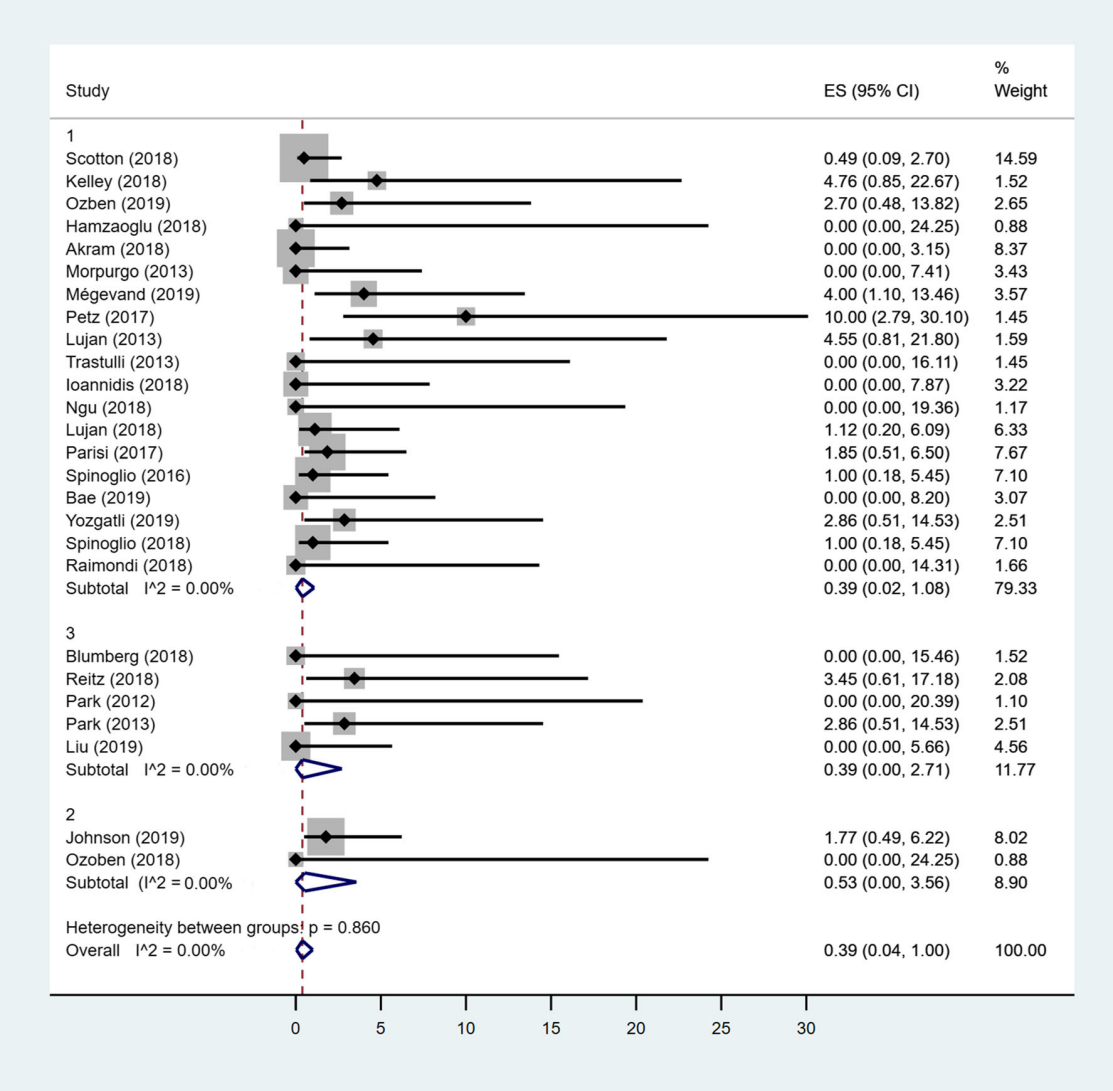
Forest plot showing bleeding rate in MD group (1), MS group (2), MSt group (3) and overall studies

Considering that in all groups, there was no heterogeneity between studies (*I*^2^ = 0); then, the fixed effects model was used to estimate the pooled prevalence. The overall pooled prevalence of bleeding was 0.39% (95% CI 0–1). The pooled prevalence for the MS group was 0.53% (95% CI 0–3.56), while the pooled prevalence among patients in MD and MSt groups was 0.39% (95% CI 0.02–1.08) and 0.39% (95% CI 0–2.71), respectively. The difference between groups was not statistically significant (*p* = 0.861) (Fig. 7).

## Discussion

From the early nineties, conventional direct manual laparoscopy had a rapid growth in several surgical scenarios, and nowadays, it is considered a cornerstone in colorectal surgery [45]. In particular, laparoscopic right colectomy has modified some traditional acquisitions; for example, it has introduced the medio-lateral dissection and the possibility to perform an extracorporeal anastomosis or an intra-corporeal one [3]. Although several studies have underlined some advantages of ICA over extracorporeal anastomosis [46, 47], it inevitably requires dexterity and more advanced laparoscopic skills, thus limiting its worldwide diffusion. In fact, in most cases, only expert laparoscopic surgeons are faced with such a technique [48]. The robotic approach, with its technological advantages, makes some surgical manoeuvres easier. In particular, thanks to these aspects and to the introduction of robotic staplers, there is a growing interest and expansion of robotic assistance during right colectomy, due to the facilitating effect that could have an impact in the reconstructive phase, contributing to the greater adoption of ICA also from less experienced surgeons. However, in spite of the increasing number of robotic systems installed, and of the increasing number of robotic right colectomies with ICA performed, it is still not sufficiently studied whether some technical details in performing the reconstruction phase robotically are superior to others.

The present review focuses on robotic ICA during right colectomy and summarises the evidence about the common techniques used. Globally, from the literature review, we found that the side-to-side, isoperistaltic anastomosis, realized with laparoscopic staplers and double-layer closure for enterocolotomy, is the most common technique used so far. However, several other variants, such as the use of robotic staplers, the totally hand-sewn anastomosis, the closure of enterocolotomy in single layer with different kind of sutures or stapled, have been significantly reported as well. Proceeding with our meta-analysis from the articles included in the review, we were able to obtain data statistically analysable within two different major groups. Thus, regarding the type of anastomosis, only for the use of robotic stapler, versus laparoscopic stapler, versus totally hand-sewn anastomosis, it was possible to obtain data to be analysed. Similarly, regarding the type of enterocolotomy closure, we could compare only the results of double layer, versus single layer, versus stapled. With respect to other further details, the heterogeneity of the studies and/or the lack of information made it possible to perform only a qualitative description.

Concerning the type of anastomosis, from the meta-analysis, we observed that the OC prevalence comparing the mechanical anastomosis with robotic and laparoscopic staplers or totally sewn, is in line with current literature regarding OC in the classic laparoscopic right colectomies [49], without obtaining though any significant difference between the three subgroups. This confirms the safety of the robotic procedure, which is comparable to the widely used laparoscopic technique, giving us also the indication that none of these different choices can be considered superior so far, with respect to the others. Also, when considering LR, we did not find any difference between the three different subgroups. However, when dealing with BR, we found in MC Rob group a reduction of BR rate respect to the others two subgroups. This may be related to the technical aspects intrinsic to robotic staplers, such as the smart clamp® technology which partially restores the presence of some ‘intelligent feedback’, by measuring the jaw closure and displaying objective feedback before firing, to optimize staple line formation. However, as far as we know, no articles in literature exist about this specific topic, and therefore, further studies are needed to investigate it and to draw conclusions.

Considering the closure of entero-colotomy, most of the works in literature report a double-layer technique, by using different sutures such as Assufil, Quill suture, Vicryl or barbed suture like V-Loc.

In apparent contrast with data from laparoscopic experience [38], the leakage and bleeding rates related to the closure of entero-colotomy in a single layer during robotic approach are not significantly higher respect to the double-layer closure. These good results of robot-assisted single layer closure respect to laparoscopy might come from the defined advantages of robotic Endo-Wrist instruments, 3D vision and suitable operative field, contributing to the general good quality of robotic suture. However, it is also possible that these similar results between SL and DL closure in robotic series are still affected by the relatively small number of procedures respect to the laparoscopic ones, and in future the superiority of DL could be assessed also in robotic procedures, with the availability of more data. Considering OC in these three subgroups, the results are again in line with current literature regarding OC in the classic laparoscopic right colectomies [49], although we noted a significant difference between the three study groups, with a lower rate of OC in MS group. However, these data seem to be not very meaningful due to the high level of heterogeneity and particularly to the great difference between the too little data on the MS group compared with the very high number of studies and therefore reported patients in the remaining two groups. Furthermore, as OC are related to several factors that may have affected the results, often beyond the mere surgical technical aspects, we think that these results should be considered a consequence of a bias. On contrary, surgical complications and in particular BR and LR are more related to intrinsic technical aspects, thereby reducing the possible impact of other variables when dealing with these aspects. In this regard, since a specific work about the enterocolotomy closure during laparoscopic fashioning of ICA in right colectomy strongly defines the superiority of DL respect to SL [38], and since the robotic assistance allows to complete DL closure without any particular effort, we think that the most cautious procedure to be adopted during robotic ICA is still the DL respect to the SL, until further solid data will be available.

Finally, it would have been useful to analyse also data about the type of suture, but unfortunately, because of the heterogeneity of the articles on this aspect, it was not possible. Thus, being quite difficult to draw definitive conclusions, we can comment this aspect only qualitatively. The safety and efficacy of barbed sutures with regards of V-Loc were just provided in recent publications for laparoscopic ICA [50, 51]. These sutures seem to be suitable during robotic assistance in which the absence of tactile feedback may limit the sutures tighten. However, the only article that extensively evaluated different ways to close the entero-colotomy during mechanical ICA after right colectomy was conducted by Milone et al. [38]. They recommended a double-layer closure using a running barbed suture in the first one. Moreover, no differences in terms of operative time and complications were noted between laparoscopic and robotic ICA. However, this multi-institutional analysis included only highly experienced centers, and this may represent a drawback.

This review has some intrinsic limitations that should be pointed out. Firstly, although we used a I2 statistic method, the studies’ heterogeneity, and the absence of all technical detailed information, limits our conclusions. Moreover, the difference in surgical robotic expertise among the included studies, as well as the absence of randomized trial, may also interfere with our results. However, to the best of our knowledge, the present article is the first to summarize different technical aspects about the use of robot during ICA in right colectomy and we found some interesting descriptive results, alongside the meta-analysis: according to available literature, the side-to-side isoperistaltic mechanical anastomosis and the double-layer closure for enterocolotomy are the wider adopted techniques for ICA fashioning during right colectomy, with a trend toward a standardized technique in clinical practice.

## Conclusions

Robotic ICA during right colectomy is a safe procedure. It can be accomplished according to several technical variants, either regarding the type of stapler used, the type of enterocolotomy closure technique, or it can be performed totally hand sewn. According to data from available literature, there is no evidence of a superiority of any of these surgical techniques respect to another. The side-to-side isoperistaltic anastomosis, realized with laparoscopic staplers and double-layer closure for enterocolotomy, is the most commonly used technique. There could be a reduction in bleeding rate with the use of robotic staplers, likely related to the technical innovations brought by the smart clamp® technology. Given the widespread of robotic surgery, and the possible impact it may have in the diffusion of ICA during right colectomy, further studies, hopefully randomized, are necessary to clarify whether a superiority may exist among one of these technical variants.

## Data Availability

All data generated or analysed during this study are included in this published article (and its supplementary information files).
